# Assessment of the Acceptability of Testing and Treatment during a Mass Drug Administration Trial for Malaria in Zambia Using Mixed Methods

**DOI:** 10.4269/ajtmh.19-0663

**Published:** 2020-06-02

**Authors:** Kafula Silumbe, Timothy P. Finn, Todd Jennings, Chilumba Sikombe, Elizabeth Chiyende, Busiku Hamainza, Elizabeth Chizema Kawesha, Thomas P. Eisele, Duncan Earle, Richard W. Steketee, John M. Miller

**Affiliations:** 1 PATH Malaria Control and Elimination Partnership in Africa (MACEPA), Lusaka, Zambia;; 2 Department of Tropical Medicine, Center for Applied Malaria Research and Evaluation, Tulane University School of Public Health and Tropical Medicine, New Orleans, Louisiana;; 3 National Malaria Elimination Centre, Zambia Ministry of Health, Lusaka, Zambia

## Abstract

From 2014 to 2016, a community-randomized controlled trial in Southern Province, Zambia, compared mass drug administration (MDA) and focal MDA (fMDA) with the standard of care. Acceptability of the intervention was assessed quantitatively using closed-ended and Likert scale–based questions posed during three household surveys conducted from April to May in 2014, 2015, and 2016 in 40 health catchments that implemented MDA and fMDA and 20 catchments that served as trial controls. In 2014 and 2015, 47 households per catchment were selected, targeting 1,880 households in MDA and fMDA trial arms; in 2016, 55 households per catchment were selected for a target of 2,200 households in MDA and fMDA trial arms. Concurrently, 27 focus group discussions and 23 in-depth interviews with 248 participants were conducted on reasons for testing and treatment refusal, reasons for nonadherence, and community perception of the MDA campaign. Results demonstrated that the MDA campaign was highly accepted with more than 99% of respondents stating that they would take treatment if positive for malaria. High acceptability at baseline could be associated with test-and-treat campaigns recently conducted in the study area. There was a large increase in the acceptability of prophylactic treatment if negative for malaria from the baseline to follow-up survey for adults and children, from 62% to 96% for each. This likely resulted from an intensive community-wide sensitization program that occurred before the first treatment round at each household during community health worker visits.

## INTRODUCTION

From December 2014 to February 2016, Zambia’s National Malaria Control Centre (renamed as the National Malaria Elimination Centre in April 2017) launched a large-scale community-randomized controlled trial to assess the impact of four rounds of community-wide mass drug administration (MDA) and focal MDA (fMDA) at the household level (fMDA), compared with a control of no mass treatment.^1^ The trial was conducted in the Southern Province of Zambia, which, according to the 2015 Zambia National Malaria Indicator Survey, had a parasite prevalence of 0.6%.^2^

The MDA arm provided dihydroartemisinin–piperaquine (DHAp) to all household residents eligible and consenting in the target health facility catchment areas (HFCAs) regardless of whether they tested positive for malaria using a standard malaria rapid diagnostic test (RDT). The fMDA arm provided treatment only to all consenting and eligible household residents if at least one person in the household tested RDT positive. Standard-of-care activities were implemented in all three arms.^3,4^ Case management included passive testing of suspected cases and treatment of confirmed or clinically diagnosed malaria cases with artemether–lumefantrine (AL or Coartem^®^, Novartis Pharma, Basel, Switzerland) at health facilities or by community health workers (CHWs). Vector control for all study arms consisted of routine distribution of long-lasting insecticide-treated nets (LLINs) and targeted use of indoor residual spraying (IRS). The control arm did not receive either MDA strategy. Extensive community sensitization activities were undertaken before and during the first and second sets of mass treatment campaigns. The community engagement strategy included district consultative meetings, local chiefs’ orientation, village meetings, drama performances, community radio messages, visually based print materials for household interactions, public address announcements, and the use of CHWs, religious leaders, teachers, and neighborhood health committees as avenues of dissemination.

The MDA and fMDA strategies provided DHAp to individuals who tested negative for malaria as well as clearance of infections in RDT-negative individuals who may harbor low-density infections that could contribute to transmission. This represented a novel approach from previous drug-based malaria interventions in Zambia, which only treated those who tested positive.^5^ As such, the trial encompassed several secondary objectives, one of which was to use a mixed-methods approach to assess the acceptability of participating in the MDA and fMDA interventions among community members in the trial arms. Acceptability in this study is defined as the extent to which people delivering or receiving a healthcare intervention, in this case malaria MDA, considered it to be appropriate based on experiences they had with the intervention.

Evidence on the common beliefs related to malaria, its prevention, and treatment that affect community members’ choices on whether or not to participate in the MDA and fMDA interventions is vital for programs to assess and modify implementation plans as needed. Previous qualitative research conducted in Zambia suggested that individuals often do not take treatment during mass campaigns because of fear of side effects, lack of perceived need (not sick), or religious objections.^6,7^

As MDA has the greatest impact when high coverage is achieved, high refusal or nonparticipation rates compromise program impact.^8^ Recently published studies have examined these issues and have reported mixed results, such as acceptability being greater when sensitization efforts are strong, individuals consistently expressing reticence and confusion over the need and purpose of blood testing during an MDA trial, and adherence to treatment courses being inconsistent and challenging to discern.^6,9–11^ Recently published qualitative work from Zambia by the same study team noted that the community as a whole accepted mass testing with treatment of malaria-positive individuals, but highlighted reasons for testing refusal.^6^ Commonly cited reasons for refusals included fear of how blood would be used (including Satanism) or, for HIV testing, not taking medication when not feeling sick, and religious beliefs.

In this article, we describe perceptions of and attitudes toward the MDA and fMDA campaigns using both quantitative and qualitative methods, with a focus on the treatment of individuals who tested negative, as well as an exploration of why individuals might not fully adhere (i.e., take all required doses correctly) to the treatment regimen. A triangulation mixed-methods study was used, consisting of a longitudinal survey with closed- and open-ended questions and community focus group discussions (FGDs) with members of nine HFCAs.

## METHODS

### Quantitative data collection and analysis.

Detailed overall study methods have been published previously (see Supplemental Appendix protocol file).^1^ In brief,^3^ household surveys were conducted from April to May in 2014, 2015, and 2016 to establish the baseline, follow-up, and final parasite infection prevalence in 40 health catchments where MDA and fMDA were implemented, and 20 catchments that served as trial controls. During these surveys, the acceptability of the interventions and community engagement were also assessed using closed-ended and Likert scale–based questions posed to individuals identified as the household head.

In 2014 and 2015, 47 households per catchment were selected from a georeferenced enumeration list by simple random samples. The sample frame comprised houses visited during previous mass test-and-treat interventions.^5^ For the acceptability research, surveyors targeted a total of 1,880 households in MDA and fMDA trial arms; because control arms did not receive MDA or fMDA, they were not assessed in this study. In 2016, the sample size was 55 households per catchment, for a target of 2,200 households in MDA and fMDA trial arms. Informed consent was sought for all participants aged 18 years or older and from the parent or guardian for all those who were younger than 18 years. Children from 6 years to 18 years provided oral assent. The household surveys were conducted pre- and post-MDA/fMDA campaign, whereas the interview/focus group survey was conducted post-MDA/fMDA campaign implementation. Ethical approval was obtained from the institutional review boards (IRBs) of Tulane University, Western IRB, the University of Zambia, and the Zambia Medicines Regulatory Authority. The full protocol for this trial, including details of the primary research questions, study design, study site, study timeline, interventions, randomization, primary outcomes, study procedures, sample size, and statistical analysis, has been published elsewhere.^1^

The survey questionnaire was based on the National Malaria Indicator Survey and modified to include modules on the acceptability of testing and treatment, as well as awareness and perception of malaria and the MDA activities.^12^ The acceptability questions sought to assess if the household head would allow a CHW to test household members for malaria, if they would take medication if positive and if negative for malaria, what the reasons were for not allowing testing or treatment, and if they would allow their children to be tested and treated if the household head refused. A series of Likert scale–based questions were read aloud to the respondents, gauging the perception of home and community-based testing and treatment. The questions were repeated during each round to ascertain differences over time. The three questions asked were as follows:

1. In your opinion, is testing and treating people with malaria in their homes a good thing for you and your family?
2. In your opinion, is it a good thing to test and provide treatment to the community in their homes to protect people from malaria?
3. In your opinion, is it a good thing to give treatment to protect people from malaria in their homes even if they are not sick?

Data were collected on personal data assistants for the baseline survey and Android mobile phones using EpiSample for the follow-up and final surveys.

Responses to binary acceptability and community engagement questions were compared across surveys to ascertain whether there were statistically significant differences in responses over time using Pearson’s chi-squared tests. Dummy variables were created to categorize responses to questions where multiple responses were allowed and analyzed across survey rounds. Robust standard errors were used to calculate CIs by including HFCA as a cluster variable. If statistically significant differences were noted, results were stratified by the trial arm (MDA versus fMDA) to assess any differences. Likert scale–based questions were stratified by baseline and follow-up survey to assess the proportion that strongly agreed, agreed, neither agreed nor disagreed, disagreed, or strongly disagreed with the statement. Responses were plotted using bar graphs to assess magnitude of responses by the survey. Likert scale–based questions were not included in the final survey in 2016. All data cleaning and statistical analyses were performed using Stata version 13.1 (StataCorp, College Station, TX).

### Qualitative data collection and analysis.

Nine health facility catchments were purposively selected from the 40 catchments in the fMDA and MDA study arms based on having high reported rates of nonadherence during the second MDA campaign round or were known to the study team to have high rates of treatment refusal. Refusal rates were used in the second round as they were noted to be slightly higher across intervention catchment areas than the first round. This outcome led investigators to use this as an opportunity to investigate reasons for refusals. A total of 27 FGDs were conducted, with three focus groups in each of the selected nine HFCAs: one group comprising eight female community members, one group comprising eight male community members, and one group comprising eight CHWs involved in the treatment campaigns of either gender. All individuals were older than 18 years, and informed consent was obtained from each participant. For logistical reasons, three interview teams conducted FGDs concurrently with a trained, experienced interviewer/moderator for each team, a note-taker, and one supervisor. Each team included at least one male and one female member. Data collection occurred from June 2015 to July 2015.

In each catchment, convenience sampling was used to identify community members who agreed to participate or who refused to participate in the intervention activities (e.g., to take the prescribed antimalarial regimens or be nonadherent) and CHWs involved in implementing the treatment campaigns. One individual was selected to be part of FGDs from every fifth house in each direction until the number of individuals satisfied the requirements of the study protocol. When the number of selected respondents who had participated in MDA reached half the number needed for the FGD, the team only recruited those who had not participated, to create an equal balance. All available CHWs in each area who participated in drug dispensing or data collection for MDA were recruited for the study. Health workers were selected based on their assigned role during the implementation of malaria MDA in districts and healthcare facilities. Standardized interview and discussion guides had been developed based on previous qualitative malaria research in this area and were adapted by the research team for the current MDA activity.^6^ Key discussion topics included community perceptions of the of the MDA and fMDA interventions, participation in the campaign and reasons for test refusal, and common reasons why community members did not adhere to treatment. The acceptability of taking medicine for malaria to clear parasites and provide prophylactic protection against malaria was a primary discussion point.

In-depth interviews (IDIs) were conducted with CHWs and health professionals drawn from the spectrum of people involved in implementation of MDA (i.e., community, facility, district, provincial, and national level). These interviews explored the key topics of community acceptance, participation, and adherence to the interventions, as well as constraints, barriers, and problems related to implementing the MDA and fMDA interventions.

In-depth interviews with catchment and district health officials were conducted in English, whereas FGDs and IDIs with community members and CHWs were conducted in the local language, Tonga. All FGDs and IDIs were audio-recorded and translated and transcribed verbatim into English. Each afternoon, fieldworkers reviewed the notes taken during the data collection exercises. These notes were transcribed or entered directly into Microsoft Word, serving as preliminary analysis reports, and were used to modify initial discussion guides. Although supervision was concurrent with fieldwork, a special meeting with the fieldworkers was held after completion of the FGDs and IDIs in each catchment to finalize all notes and documents and to provide a summary report.

Field reports from qualitative teams were reviewed to determine overall themes of each FGD and IDI. Data analysis followed a three-stage approach. First, the transcripts were read twice, line by line, noting key discussion points and compared with field reports to confirm consistency. Second, using the results of the quantitative acceptability questions and the interview topic guides, an a priori code book was developed around the three primary discussion points. Last, transcripts were read in NVivo (QSR International Pty Ltd., Melbourne, Australia) and codes were applied. Where new concepts were noted, additional codes were created in NVivo. Coding and analysis of transcripts was performed in NVivo 11 (NVivo qualitative data analysis software, QSR International Pty Ltd. [Melbourne, Australia] Version 11.4, 2016).

## RESULTS

### Quantitative results.

Household surveys targeted 1,880 households for baseline and follow-up and 2,220 for the final survey. A total of 1,707 (91%), 1,518 (81%), and 1,851 (83%) households, respectively, participated in baseline, follow-up, and final surveys. The mean age of respondents was 45 years, with 77% being male. Tables 1–3 present the survey results. At each survey, there were no differences in results by trial arms (results not shown). Acceptability of testing was nearly universal across each survey at 98% or greater with no statistically significant differences. Self-reported acceptability of treatment based on RDT-positive tests showed a significant increase (*P* = 0.02) from baseline at 93% (95% CI = 0.86–0.97) to final at 99% (95% CI = 0.98–0.99). Acceptability of treatment for RDT-positive children was nearly universal among respondents at 99%. Results were slightly less at follow-up but similarly high at 97%. Regarding acceptability of taking prophylactic treatment when testing negative, two questions were posed: one addressing the respondent and one addressing their children. For self-reported acceptability and for acceptability of having one’s children testing negative for malaria, the proportion that would accept preventive treatment increased significantly from the 2014 baseline at 62% (95% CI = 0.51–0.73) to 98% (95% CI = 0.97–0.99, *P* < 0.001) and 97% (95% CI = 0.95–0.98, *P* < 0.001), for the 2015 and 2016 surveys, respectively.

**Table 1:** Acceptability of testing and treatment by survey for malaria mass drug administration–implementing areas in southern Zambia in 2016

Question	Baseline		Follow-up		Final		*P*-value
	*n*	Prop (95% CI)	*n*	Prop (95% CI)	*n*	Prop (95% CI)	
Would allow MoH worker to test respondent and children for malaria	1,646	0.98 (0.97–0.99)	1,502	0.98 (0.97–0.99)	1,828	0.99 (0.98–0.99)	0.49
Would take malaria treatment if tested positive for malaria	1,664	0.93 (0.86–0.97)	1,502	0.96 (0.92–0.98)	1,814	0.99 (0.98–0.99)	0.02*
Would allow children to take malaria treatment if they tested positive for malaria	1,668	0.99 (0.97–0.99)	1,502	0.97 (0.93–0.98)	1,796	0.99 (0.98–0.99)	0.001†
Would take prophylactic malaria treatment if tested negative for malaria	1,669	0.62 (0.51–0.73)	1,502	0.95 (0.92–0.97)	1,800	0.98 (0.97–0.99)	0.001†
Would allow children to take prophylactic malaria treatment if tested negative for malaria	1,667	0.62 (0.51–0.73)	1,460	0.96 (0.94–0.98)	1,806	0.97 (0.95–0.98)	0.001†

* Significant difference at *P* < 0.05.

† Significant difference at *P* < 0.001.

**Table 2:** Community awareness of malaria for malaria mass drug administration–implementing areas in southern Zambia in 2016

Question	Follow-up		Final		*P*-value
	*n*	Prop (95% CI)	*N*	Prop (95% CI)	
Malaria is still a problem in community (yes)	1,471	0.44 (0.34–0.55)	1,784	0.40 (0.30–0.51)	0.55
Rank of malaria as a health problem in the community	1,503		1,811		0.31
Very high		0.02 (0.01–0.03)		0.01 (0.00–0.01)	
High		0.09 (0.06–0.14)		0.06 (0.04–0.10)	
Moderate		0.22 (0.17–0.28)		0.20 (0.15–0.27)	
Low		0.64 (0.57–0.71)		0.70 (0.62–0.77)	
Not a problem		0.03 (0.02–0.06)		0.03 (0.02–0.04)	
Amount of malaria in the community in the past 12 months	1,503		1,811		0.14
More		0.09 (0.06–0.13)		0.06 (0.03–0.09)	
Less		0.87 (0.83–0.91)		0.90 (0.86–0.93)	
The same		0.04 (0.02–0.06)		0.04 (0.03–0.06)	

**Table 3:** Post-MDA community perception and engagement

Question	Follow-up		Final		*P*-value
	*N*	Prop (95% CI)	*n*	Prop (95% CI)	
Heard about the MoH MDA program (yes)	1,491	0.71 (0.65–0.76)	1,764	0.74 (0.66–0.80)	0.33
How they learned about the campaign	1,061		1,311		
Community (neighbor and leader)		0.30 (0.25–0.37)		0.28 (0.22–0.35)	0.58
Media		0.19 (0.15–0.25)		0.16 (0.13–0.19)	0.17
Health system		0.70 (0.62–0.76)		0.75 (0.70–0.80)	0.24
Why they participated in the MDA campaign	1,058		1,305		
Told by CHW		0.11 (0.07–0.16)		0.09 (0.06–0.14)	0.64
Concerned about family		0.60 (0.52–0.67)		0.66 (0.59–0.72)	0.28
Protect community from malaria		0.40 (0.33–0.47)		0.44 (0.36–0.52)	0.52
Trust MoH		0.08 (0.05–0.13)		0.10 (0.07–0.14)	0.64
Did not participate		0.20 (0.15–0.27)		0.13 (0.09–0.20)	0.08
What participants liked about MDA	844		1,129		
CHW knowledgeable, trustworthy		0.09 (0.05–0.16)		0.05 (0.03–0.10)	0.24
Convenience (at home, free)		0.79 (0.73–0.84)		0.85 (0.80–0.89)	0.09
Easier treatment than Coartem		0.10 (0.06–0.14)		0.09 (0.06–0.13)	0.81
Prophylaxis for a month		0.31 (0.23–0.40)		0.33 (0.23–0.43)	0.77
What participants did not like about MDA	829		1,130		0.27
CHW unknown, not friendly		0.01 (0.01–0.03)		0.02 (0.01–0.04)	0.27
Poor timing (rainy season, took too long)		0.26 (0.17–0.37)		0.17 (0.11–0.26)	0.16
Treatment unfamiliar		0.05 (0.03–0.08)		0.15 (0.09–0.22)	0.00*
Pills tasted bad, bitter		0.04 (0.02–0.11)		0.07 (0.04–0.12)	0.38
Drugs made them feel sick, side effects		0.30 (0.22–0.39)		0.20 (0.15–0.28)	0.09
Tired of testing and treatment visits		0.01 (0.00–0.02)		0.02 (0.01–0.03)	0.12
Would participate in future MDA campaigns if visited again	1,503		1,817		0.03*
Yes		0.87 (0.80–0.92)		0.94 (0.91–0.96)	
No		0.08 (0.04–0.14)		0.02 (0.02–0.04)	
Do not know		0.05 (0.02–0.11)		0.03 (0.02–0.07)	
What it would take to participate in future MDA campaigns	121		50		
Better sensitization		0.92 (0.79–0.97)		0.78 (0.65–0.87)	0.07
More knowledgeable CHW, CHW from community		0.95 (0.85–0.99)		0.92 (0.82–0.97)	0.55
Will not participate		0.57 (0.33–0.78)		0.28 (0.14–0.48)	0.04*

CHW = community health worker; MDA = mass drug administration.

* Significant difference at *P* < 0.05.

Table 2 presents results of community awareness questions posed at the follow-up and final surveys. There was a slight decrease in the proportion of respondents stating that malaria was still a problem in the community, and there was a shift in the distribution of responses from a high and moderate ranking of malaria as a problem in the community to moderate and low, respectively, with marginal statistical significance.

Table 3 presents information on how households became aware of the MDA campaigns, whether they participated, and, if so, what they liked and disliked about the campaign. Respondents were able to choose multiple responses. In each survey, more than 70% had heard about the campaign, with the majority hearing from CHWs or the clinic staff, followed by neighbors or community leaders, and, last, media. Most respondents stated that they participated because of concern about their family’s health and to help the community protect itself from malaria. Twenty percent of respondents in the final survey had not participated in the MDA. Awareness of the prophylactic benefits of MDA was consistent between surveys in one-third of respondents. Respondents claiming that they were unfamiliar with treatment increased significantly (*P* < 0.05) from 5% (95% CI = 0.03–0.08) to 15% (95% CI = 0.09–0.22) in the final survey. There was a 10% decline in those noting that DHAp caused side effects, from 30% to 20%.

**Figure 1.**
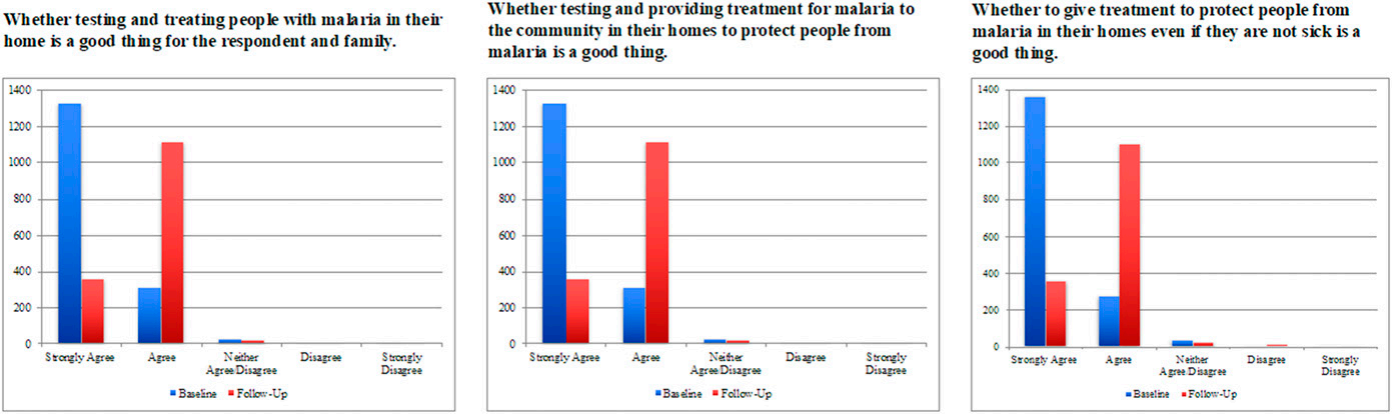
Responses to Likert scale–based questions from baseline to follow-up surveys for malaria mass drug administration–implementing areas of southern Zambia. This figure appears in color at www.ajtmh.org.

Those that did not participate in the preceding MDA round were asked what it would take for them to participate in an MDA campaign. Figure 1 presents results from Likert scale–based questions. Responses were consistent for each of the three questions posed at baseline and for the follow-up survey. The results indicated that people generally agreed with the idea of being provided with testing and treatment at their homes and for prophylaxis. Notably, of 1,669 respondents at baseline, 62% noted they would take prophylactic malaria treatment if they tested negative for malaria, whereas of 1,800 respondents following the final survey, 98% responded they would take prophylactic treatment. Similarly, when asked if they would allow children to take prophylactic malaria treatment if they tested negative for malaria, 62% and 97% of respondents at baseline and during the final survey, respectively, noted that they would.

### Qualitative results.

A total of 27 FGDs and 23 IDIs with 248 participants were conducted. Table 4 outlines the participation by catchment. The FGDs with community members centered on three main discussion areas: reasons for testing and treatment refusal, reasons for nonadherence, and community perception of the MDA campaign. Approximately halfway through the data analysis, data saturation was reached in relation to FGDs after 12 FGDs were coded and no new themes or reasons were noted.^13^ Similarly, roughly halfway through the data collection, saturation was reached for the IDIs. A total of 27 FGDs and 21 IDIs were coded in NVivo.

**Table 4:** Malaria MDA review of qualitative survey sampling for the implementing areas in southern Zambia in 2016

District	Catchment	Arm	IDIs (*n*)	FGDs (*n*)	Male FGD	Female FGD	CHW FGD	
					Men (*n*)	Women (*n*)	Men (*n*)	Women (*n*)
Chikankata	Cheeba	fMDA	3	3	8	8	5	3
Gwembe	Sinafala	MDA	1	3	8	8	4	4
Gwembe	Luumbo	MDA	2	3	8	8	5	4
Kalomo	Kanchele	fMDA	1	3	8	8	7	4
Kalomo	Dimbwe	MDA	4	3	8	8	7	5
Siavonga	Matua	MDA	2	3	8	8	6	4
Sinazongwe	Buleyamalima	MDA	4	3	8	8	5	4
Zimba	Mapatizya	MDA	2	3	8	8	7	6
Zimba	Luyaba	MDA	2	3	8	8	6	5
Total			21	27	72	72	52	39

CHW = community health worker; fMDA = focal MDA; FGD = focus group discussion; MDA = mass drug administration.

### Acceptability and perception of RDT testing.

The campaign was well received overall by participants, but a clear consensus formed regarding RDT testing in the community. The proportion of reported refusals based on the intervention data was less than 1% overall in all MDA rounds. Community members who refused stated that they did so because of concerns whether CHWs or the program was tenets of Satanism, concerns that their blood would be used for HIV diagnosis, dislike of some of the side effects associated with DHAp, and, generally, inadequate knowledge about the MDA campaign and treatment. It was noted frequently that these individuals were not adequately sensitized to the program, the reason why blood would be tested, and why individuals who were RDT negative would be provided treatment. This lack of awareness abated over time but initially characterized the rollout of the intervention during rounds 1 and 2.

Community health workers and health officials validated these assertions in FGDs and IDIs. As one CHW noted:

“We tried to teach them [but some] were saying this medicine you have brought is Satanic. Others would say they are ARVs [antiretroviral].…But in the first round the majority got [treated] and second round those who refused in the first round agreed after seeing the goodness of the medicine.” (Cheeba, CHW)

Some initial concerns stemmed from misunderstandings about the nature of the MDA trial in an area of Zambia that has had a number of programs that used blood tests for HIV-testing programs and where mass distribution of medications for lymphatic filariasis and trachoma has been conducted.

### Perception of DHAp treatment when RDT negative.

Many of the areas that participated in the MDA trial also participated in a test-and-treat trial in 2012–2013 and had an active CHW-led case response program that used Coartem^®^ for treatment of malaria-positive individuals.^5^ Thus, the MDA trial presented a marked shift in the treatment strategy with the treatment of all individuals with a new medication, DHAp, regardless of test positivity. Participants in the FGDs initially expressed confusion about this shift in strategy because of lack of community sensitization.

“It was just difficult [to grasp] that even when you are not sick, you were supposed to take the pills when they teach us at the clinic that we are only supposed to take drugs when you are sick. Just like I can’t go to the clinic and [request] to be given drugs [if not ill], it was difficult for others to accept taking medicine when they were not sick.” (Luumbo, male)

Yet, as the program proceeded, in areas where sensitization was not cited as being a factor for lack of awareness, individuals noted the distinction between treatment and prevention, awareness of the prophylactic benefits of DHAp, and the concept of asymptomatic infections or “hidden malaria”:

“The one who tested me explained what they were doing; they said they were testing for malaria, saying that if they find you positive or negative they still gave medicine. For the positive, it was treatment and for the negative it was prevention. I think a person must explain the program very well so people can understand.” (Matua, male respondent)

“We feel good because when they have tested me, the malaria parasite will not be strong to fight my body. Nowadays I really feel free because I know the parasite will be dealt with even before I get sick.” (Mapatizya, female respondent)

### Adherence to DHAp treatment.

There was a consensus among all groups that some people stopped taking the medication after the first dose after experiencing vomiting or diarrhea, or hearing rumors about side effects. These findings are consistent with the explanations provided by respondents during the quantitative questionnaire that stated why they refused or did not finish the treatment.

“The reason why most people stopped taking the medicine was because of the report they received from the other people who took it and had side effects…that was the major challenge we had in February [round 2].” (Matua, CHW)

The role of adherence officers who followed up DHAp treatments on the third day was crucial in ensuring treatment adherence. Some participants, as highlighted in the following paragraphs, cited adherence officers as a reason for finishing treatment courses as the adherence officers encouraged individuals by following up directly and reminding them to take their medication.

“Those problems used to be there. [Some people] would take the medication [and] maybe they vomit [or] they have dizziness. Depending on the different side effects, others just end up deciding to just stop. But when the adherence officers follow up in day 3, they advise them to finish off with the medication.” (Dimbwe, CHW)

### Perceived impact of MDA.

With respect to community perception of MDA, FGD participants revealed that community members were generally pleased with the MDA campaign. They noted that DHAp was more effective than Coartem^®^ and had a reduced pill and dosage burden. Many individuals noted not experiencing a malaria episode since completing the medication. This view was also expressed by CHWs and health officials during IDIs.

“Before this medication was introduced, most people never used to stay [malaria-free] for a long period of time without getting sick but now this drug has helped us stay [healthy] for a very long time without malaria. Most of us have stayed [healthy] since we took the medication up to now without getting sick of malaria so this medication has helped us in a good way.” (Luumbo, male respondent)

In addition, after MDA with DHAp, there were fewer cases at the clinic than in previous years, suggesting a general decline in prevalence.^1^

“This program must continue because we get treated before we get sick. Secondly, it has reduced the congestion at the clinic in terms of malaria cases.” (Kanchele, male respondent)

Last, demonstrating the knowledge of the goal of the program to eliminate malaria in Zambia, opinions differed, but there was, again, consensus on the impact of MDA in the short term.

“Many people who took the medicine according to the instructions had malaria wiped out of their bodies; [for] others, it was still there. So I think if we continue, malaria will end in Zambia.” (Mapatizya, CHW)

“From the time we took the malaria drugs, there has been change as compared to before we started taking the drugs. The number of people who get sick from malaria has reduced; I cannot really say that these drugs have eliminated malaria because it cannot completely finish, but I can say there is change.” (Buleyamalima, male respondent)

### Community suggestions for future MDA.

The need for more intensive sensitization was expressed by everyone. There was an inconsistent rollout of the sensitization campaign in larger health catchments because of limited road accessibility and a lack of radio signal coverage. The use of headmen to inform community residents appeared to have mixed results. However, participants noted that increased exposure to the program addressed many of these issues.

## DISCUSSION

The evidence from this mixed-methods assessment of MDA acceptability demonstrated that MDA was generally accepted. The willingness of individuals to take treatment if found RDT negative is crucial for the long-term effectiveness of the MDA strategy. Encouragingly, results indicate that there was a substantial increase in acceptability of prophylactic treatment for those found RDT negative for malaria from the baseline to follow-up survey; indeed, at the final survey, acceptability for this strategy was more than 97% for both adults and children. This likely resulted from the intensive community-wide sensitization program that occurred before the first treatment round and at each household during CHW visits. Findings from the community FGDs corroborate this increase in acceptability of prophylactic treatment; many participants stated that those who were RDT positive noted the curative effect of the treatment and those who did not have malaria understood the preventive component and noted not having another infection in the months after the campaign. However, the study did not explicitly collect data on understanding the role of asymptomatic infections in malaria transmission.

There was no difference in acceptability of testing and treatment for RDT positives, that is, nearly everyone who agreed to be tested also accepted malaria treatment. This was in line with expectations for the study area. Many of the trial catchments participated in a mass testing and treatment campaign with AL in 2012 and had a robust community-led malaria control program with high levels of LLIN usage, IRS, and CHW case management coverage.^5^ Fear of side effects and the use of blood for Satanism were often cited as barriers to individual acceptability of testing and adherence. These results are not dissimilar from the results of previous qualitative work carried out in the same area after the mass test-and-treatment campaign.^6^ In a similar qualitative study that examined pre- and post-community acceptability of a trial of mass testing and treatment of only RDT-positive individuals with DHAp in Kenya, FGD participants expressed parallel concerns about the use and disposal of blood during testing, high concordance with taking treatment if positive, the effectiveness and easier dosing of DHAp compared with Coartem, and mixed results regarding treatment adherence.^9^

Fear of side effects was enhanced by hearsay from neighbors or the community and experiencing mild side effects such as dizziness or diarrhea, which are commonly associated with DHAp treatment.^14^ These perceptions may have contributed to individuals not completing the treatment regimen or keeping the medication for later use. Indeed, some reported that they kept the medication noted subsequently taking it after learning that it was safe. It is not possible to quantify how pervasive this phenomenon was during the first two MDA rounds to assess the reliability of the reported adherence data collected by adherence officers. Survey data on sensitization and participation were not linked to individual adherence data during the trial, thus limiting the ability to discern the degree and method of community sensitization among individuals who did not adhere to the treatment or refused testing. The reasons provided for nonadherence were primarily reported as forgetting to take doses, losing medication, and feeling better, with side effects cited by only 5% of participants. These results were comparable with a study in Kenya by Shuford et al.,^9^ where individuals highlighted concerns over side effects, discontinuing treatment after feeling better, and a discordance with understanding why they should take treatment but failing to do so. Given the growing body of evidence that suggests individuals may not be fully adherent, MDA implementers must work to ensure that before the initiation of campaigns, clear messages regarding the importance of completing treatment regimens are delivered and embraced by the community. It is advisable that intense and consistent community engagement should be prescribed for a prolonged period of time before and during implementation.

Community health workers were generally praised for their conduct, but in certain areas where previous test-and-treat campaigns provided treatment only to positive individuals, community members were understandably confused about why they were provided medication if found to be RDT negative. Although recognition of the prophylactic benefits of DHAp treatment provided to test-negative individuals increased over time, the rollout of the campaign’s first two rounds was affected by initially heterogeneous coverage of sensitization activities. In this campaign, efforts at community sensitization involved community meetings with chiefs and headmen and the use of radio and mobile broadcast trucks. However, as noted in the FGDs, not all areas received radio signals or were accessible by vehicles, and headmen did not consistently inform residents of the campaign’s purpose and benefits. In a recent but comparatively small study examining participation in MDA administration with DHAp in Vietnam, Nguyen et al.^10^ found that individuals reporting full adherence to DHAp noted receiving sensitization from the district health teams. Moreover, in a comparable trial along the Thai–Myanmar border, researchers found that nonparticipation in MDA was related to not understanding the nature of the intervention.^15^ Areas remote from clinics require greater investment in liaising with community leaders and/or activities led by designated individuals who are recognized members of the community health structure, so community members can be properly sensitized in advance of campaigns.

These general issues expressed by many FGD participants reiterate the need for large-scale programs that may conduct blood testing and/or provision of treatment to noninfected individuals to focus on intensive community sensitization for a prolonged period before the campaigns begin. Although no person should be compelled to take medication against their beliefs, consistent reports of pockets of communities that do not participate in scaled malaria elimination intervention efforts may require more focused community-level health education with an emphasis on the use of and the provision of LLINs and implementation of targeted IRS as Kajeechiwa et al.^15^ also had stated.

Last, as one prescient FGD participant stated:

“Okay, I have heard what you have said that the drugs were for protection and cure, but my next question is: why did they used to test first since whether they found you with malaria or not they would still give you the drugs? What was the point of testing if at the end of it all they would still give you the drugs?” (Buleyamalima, male respondent)

Mass drug administration programs for malaria should examine whether large-scale RDT testing is necessary given continued concerns over blood testing in the setting where treatment will be provided to everyone. Understanding prevalence may be less integral to program effectiveness if fear of blood testing detracts from an enhanced focus on emphasizing the importance of taking all doses. This is partly why the National Malaria Elimination Centre phased out testing in the latest programmatic rounds of MDA in 2017 and 2018.

Our study had some limitations. First, convenience sampling was used to recruit community members for the FGDs. Because individuals who refused testing and treatment were unlikely to participate, community members and CHWs provided secondhand information about refusals. This may have resulted in biased responses that may not have accurately reflected reasons for testing and treatment refusal. However, as noted, refusals reported by CHWs were low overall, and FGD findings were comparable with the quantitative acceptability results. Second, data were collected at different time points, which could be a potential source of bias as respondents might give different responses based on whether they participated in an IDI or FGD first. Third, a limitation of the qualitative method was that it was not able to gather information for all CHWs involved in MDA campaigns to make it even more representative. Limitations of the quantitative method were that only a select few persons in each household (i.e., head of household and women of child bearing age) were interviewed. Furthermore, the catchments participating in the qualitative component of this study were purposively selected because of known implementation issues and reports that refusals were high and adherence was low. Fourth, a delay in trial implementation meant that there was a time gap between sensitization efforts and the rollout of the intervention. Finally, the main trial did not collect awareness and perception of malaria as a community health problem at baseline.

Overall, these findings indicate that the MDA campaign was highly accepted and perceived as a valuable intervention resulting in the reduction of the malaria burden. Participants noted that DHAp was well tolerated and that they experienced fewer episodes of malaria.^16^ Targeting appropriate health education to individuals who consistently refuse testing and treatment because of fear of Satanism or privacy concerns about HIV remains a challenge, however. If such beliefs persist and are widespread, programs will need to ensure these community members have access to all other malaria control and prevention services, as participation in MDA campaigns is voluntary. Evidence that malaria is now perceived to be a lower health priority in the communities studied is a testament to the efforts of National Malaria Elimination Centre and partners in scaling up malaria prevention and control services in this area over the last decade.

As noted, future MDA campaigns should consider removing the RDT testing component, and an alternative means of assessing program impact should be identified. Alternatively, testing could be optional and treatment provided regardless of RDT results, although interpretation of such data as a reflection of community infection distribution would present some challenges. The concerns expressed regarding Satanism associated with the MDA campaigns suggest that local church leaders should be included in sensitization efforts to dispel myths concerning interventions and facilitate participation. Provision of medication to individuals without symptoms or a positive RDT test result requires intensive sensitization in advance of household visits to explain the rationale for treatment and full adherence. MDA programs must monitor community uptake of the intervention and full adherence to the treatment regimen. Adherence follow-up visits, although labor intensive, may have beneficial impact on reducing concerns over minor side effects, clarifying drug dosing and improving full adherence. As malaria cases decrease and community perception of malaria as a health problem declines, programs must continue intensive education and reinforcement of the importance of continued community engagement with prevention and control measures.

## RECOMMENDATIONS

Perception of malaria as a lower health priority is a sign that the work is progressing well, but it also means repeat sensitization campaigns will be needed to ensure that MDA and fMDA campaigns are not seen as “irrelevant” and, therefore, have low compliance rates leading to a resurgence of malaria in the future.

## Supplemental appendix file

Supplemental materials
