# Supplementary material for: Assessment of the Acceptability of Testing and Treatment during a Mass Drug Administration Trial for Malaria in Zambia Using Mixed Methods
Source: Am J Trop Med Hyg. 2020 Jun 2;103(2 Suppl):28–36. doi: 10.4269/ajtmh.19-0663 (PMC7416978; doi:10.4269/ajtmh.19-0663)
Supplement: Supplementary file 1 [file tpmd190663.SD1.doc]

Supplemental Appendix 13 – Group qualitative interview discussion guides (rural health centers, district health teams, national malaria control center personnel and ministry of health officials)

Version: 16 July 2012

Purpose:

The group interviews are designed to answer the following questions:

1. How is the Mass Screen and Treat (MSAT) campaign is affecting community health workers?

2. Can the MSAT campaign be scaled beyond the current target districts, and what would be required to do so?

3. Has the increased malaria surveillance data influenced the behaviors and perceptions of the health worker, and if so how?

Group discussion guide:

What do you think about the test and treat campaigns in Southern Province?

Is the test and treat campaign something that you might consider applying in other areas?

What makes it appealing?

What makes it unappealing?

Would you conduct the campaigns any differently?

What changes might you make and why?

Have the campaigns had any other influence other than the actual intervention being conducted?

What do you think about the data coming back from the campaigns?

Has this data influenced your decision-making?

How are the campaigns affecting the community health workers' regular duties?

What do you think about the data coming back from the cell phones in rural health centers?

Has this data influenced your decision-making?
