# Supplementary material for: Assessment of the Acceptability of Testing and Treatment during a Mass Drug Administration Trial for Malaria in Zambia Using Mixed Methods
Source: Am J Trop Med Hyg. 2020 Jun 2;103(2 Suppl):28–36. doi: 10.4269/ajtmh.19-0663 (PMC7416978; doi:10.4269/ajtmh.19-0663)
Supplement: Supplementary file 2 [file tpmd190663.SD2.docx]

**Supplmental Material**

**Protocol: Acceptability of DHAp in the community**

***Implementation procedures***

The assessment of community acceptability of the fMDA and MDA interventions will be ascertained using a mixed methods approach. Acceptability of the interventions will be assessed quantitatively during the baseline and follow-up parasite surveys among women of reproductive age (WRA). Focus groups will also be used to qualitatively assess community perceptions of the acceptability of the fMDA and MDA interventions, including community members who consented and participated as well as those who refused. Within both groups, perceived barriers to participating in the interventions will also be assessed. In particular, the acceptability of taking medicines for malaria to clear parasites and provide prophylactic protection against malaria among those in the MDA intervention will be assessed quantitatively and qualitatively. Focus group and semi-structured interviews of community health workers (CHWs) will be used to assess barriers to adherence, experience in implementing the interventions, and the acceptability of the interventions among community members.

***Primary outcomes***

The primary qualitative outcome for this study will be the common perceived beliefs on why community members choose to participate and choose not to participate in the fMDA and MDA interventions. The primary quantitative outcome will be the proportion of survey respondents who strongly disagree, disagree, are ambivalent, agree, and strongly agree on the importance of community mass treatment and the provision of prophylactic protection.

***Sample size***

For the primary quantitative indicator above, the sample of 2,700 households across the fMDA and MDA interventions at the baseline (2014) and again at follow-up (2015) survey rounds will be used. This is expected to provide a sample size of at least 4,000 individuals at each round, which is sufficient to estimate the proportion of participants who agree that the MDA and fMDA intervention are important (agree and strongly agree) with ±5% intervals with the probability of committing a type-1 error of 5% (1-tailed test).

For the qualitative indicator above, convenience sampling will be used to 1) identify community members in the fMDA and MDA intervention areas who agreed to participate and who refused to participate in the 2014 and 2015 fMDA and MDA interventions (e.g. to take the prescribed antimalarial regimens) and 2) community health workers and other health program staff implementing the interventions.

Approximately 100 individuals in the fMDA and MDA interventions (50 in the fMDA and 50 in the MDA during the 2014 and 2015 rounds) will be targeted for qualitative data collection using focus group interviews. Approximately 30 individuals identified as CHWs or health facility staff will additionally be targeted for qualitative data collection during focus group interviews. A total sample size of 130 men and women ≥18 years old will be targeted in 2014 and again in 2015 (total n = 260).

***Data collection***

Informed consent will be obtained to participate specifically in the semi-structured and focus group interviews. It is expected that five focus groups of ten community members will be conducted in the fMDA (n = 5 groups and 50 community members) and MDA (n = 5 groups and 50 community members) groups after the third fMDA/MDA round in each year, totaling 200 community members. Standardized interview and discussion guides will be established using pre-testing and best practices in qualitative research. Trained interviewers will be used to conduct the interviews in the local languages. An additional two focus groups of ten community health workers will be conducted after the third survey round in each year (n = four groups and 40 health workers).

Semi-structured interviews will be conducted with community health workers and other health professionals who implemented the interventions. These interviews will explore constraints and problems related to all the topics identified from the quantitative survey, field experience implementing the fMDA and MDA interventions, and barriers to implementation of malaria control efforts. They are also important in order to involve providers in the study.

A specific acceptability module with Likert scale questions will be added to the Parasite Survey questionnaire and collected as part of the Parasite Surveys in 2014 and 2015, as described in detail above.

***Analytic plan***

The interviews will be conducted in local languages and field notes will be written during the interview. Each afternoon, fieldworkers will return to a central location and work with the local investigator to systematically record field notes in English (using the ‘Fair Notes’ data capture method). These notes will be transcribed or entered directly into MS Word. dtSearch 6.0 © (dtSearch Corp.) will be used to index and search these notes as data collection proceeds. Analysis will identify thesauri of common themes and interpretations and will summarize each section of the assessment guide. These summaries will be compared to summaries maintained by the fieldworkers. Although supervision will take place each day, a special meeting with the fieldworkers will be held to review findings from data and to follow up with special issues.

Pile sorting of responses to questions surrounding the testing and treatment of adults and children will be performed for group interviews, with most common and important barriers to fully participating in the fMDA and MDA interventions identified.

For the quantitative analysis, Likert scale data will be grouped as needed using Cronbach’s coefficient alpha. Data will then be categorized and analyzed using descriptive statistics, χ2s and logistic regression.
