# Supplementary material for: Assessment of the Acceptability of Testing and Treatment during a Mass Drug Administration Trial for Malaria in Zambia Using Mixed Methods
Source: Am J Trop Med Hyg. 2020 Jun 2;103(2 Suppl):28–36. doi: 10.4269/ajtmh.19-0663 (PMC7416978; doi:10.4269/ajtmh.19-0663)
Supplement: Supplementary file 3 [file tpmd190663.SD3.docx]

Supplemental Appendix 6 – Individual and group qualitative interview discussion guides

Version: 16 July 2012

**Research Questions:**

1. How do people feel about repetitive malaria infection screening and treatment and what are possible reasons people may refuse the intervention?

2. Are people testing positive complying with treatment regimens, and what are potential reasons for treatment non-compliance?

3. How is the MSAT campaign affecting community health workers?

4. Can the MSAT campaign be scaled beyond the current target districts, and what would be required to do so?

5. Has the increased malaria surveillance data influenced the behaviors and perceptions of the health worker, and if so how?

**Interview guide for individuals receiving the intervention:**

**(Research questions 1, 2)**

I’d like to start our discussion by getting to know you a little bit better, and what kinds of health issues are important where you live.

What are the major health concerns in your community?

What are the major health concerns in your family?

What kinds of things do you do to stay healthy?

What do you typically do if someone in you or someone in your family gets sick?

As I mentioned before, one of the health issues we are interested in learning more about is Malaria. What have you heard about Malaria?

Have you or anyone in your family ever gotten sick with Malaria? Tell me more about that…

Tell me about what happened when you had malaria, from the time you suspected you had it until you got better.

What is it?

How do people get infected with malaria?

How can you prevent malaria infection?

How can you treat malaria infection?

Have you ever been ill with malaria?

When was that?

Did you seek treatment or do anything for the illness?

How can someone know if they have malaria?

When was the last time you were tested for a malaria infection?

Tell me about how you ended up testing for malaria…

Were you ill at the time?

Who tested you?

Did they test other people in your family for malaria infection?

Did you test positive or negative?

Did anybody in your family test positive?

What are the chances that someone could test positive for malaria, but not feel sick?Tell me more about that…

Who would you suggest test for malaria?

Tell me about the kinds of things people can do/should do to get better when they have malaria.

What about medicine for malaria? What different types of medicine have you heard about? What type of medicine do you get from a health centre?

What do people do with the medicine they get from the health center?

Should they receive medicine?

What should they do with the medicine they receive?

The treatment for malaria infection is 3-days worth of pills. Do people finish the medicine?

Should they save the medicine?

Should they trade or sell the medicine for something they need?

What if they feel better before all the medicine is gone?

Did you receive any medicine when you were tested for malaria infection?

What did you do with the medicine?

Did anyone in your family receive medicine when they were tested for malaria infection?

What did they do with the medicine?

What do you think about testing people for malaria infection when they are not sick with symptoms?

What do you think about people receiving medicine for malaria infection when they test positive even if they are not sick with symptoms

Have you ever chosen not to test for malaria when you were offered the test?

If so, what were the circumstances?

If not, would you ever consider refusing a test for malaria infection?

Have you ever chosen not to test your child for malaria infection when they were offered the test?

If so, what were the circumstances?

If not, would you ever consider refusing your child being tested for malaria infection?

Do you know anybody who refused being tested for malaria infection, or who refused his or her child being tested for malaria infection?

Why might someone refuse a malaria infection test?

Have you ever refused treatment for a malaria infection?

If so, what were the circumstances?

If not, would you ever consider refusing treatment for a malaria infection?

Have you refused treatment for a malaria infection for your child?

Have you ever refused malaria treatment for your child?

Have you ever saved or traded malaria treatment meant for your child?

What do you think about the campaign to screen everybody in your community for malaria infection?

What do other people think about the campaign?

What kinds of things are people saying about the campaign?

Are people in your community refusing being tested for malaria infection?

What are some of the reasons someone might choose not to test for malaria?

Are people in your community finishing their medicine when they test positive for malaria infection?

Why might someone not finish their medicine when they test positive for malaria infection?

As I mentioned at the beginning of the interview, one of the reasons we are doing interviews with people in your community is to learn more about how to improve testing campaigns and malaria services.

One of the problems is that sometime people do not want to test for malaria. What is your view on why this might be happening? What do you suggest that could be done so that more people might want to test?

Another question we are trying to answer, is how to help make sure people get the treatment that they need to get better when they have malaria. Sometime, people don’t take all their medicine, even when they get it from a health provider. Tell me what you’ve heard about this. What is your opinion about why this might be happening? What do you suggest that could be done to support people taking all their medicine?

Do you think it is good to have community health workers (CHWs) testing in the community?

Is your CHW doing a good job? What would you change?

**Interview guide for individual interviews with community health workers:**

**(Research questions 1, 2, 3, 4. 5)**

I’d like to start off by getting to know you a little bit better. Tell me about how you ended up working in malaria prevention and treatment?

-How long have you provided services for malaria?

-What things do you like about working in malaria?

-What things would you change about your job?

-How did you hear about the malaria testing campaign ?

-How would you compare your job working in the campaign to your regular job as a health provider? What types of things do you like better about working in the campaign? What types of things are more difficult, working in the campaign versus your regular job.

What do you know about malaria?

What is it?

How do people get infected with malaria?

How can you prevent malaria infection?

How can you treat malaria infection?

Have you ever been ill with malaria?

When was that?

Did you seek treatment or do anything for the illness?

When was the last time you were tested for a malaria infection?

Were you ill at the time?

Who tested you?

Did they test other people in your family for malaria infection?

Did you test positive or negative?

Did anybody in your family test positive?

What do you think about testing people for malaria infection when they are not sick with symptoms?

What do you think about people testing positive for a malaria infection when they are not sick?

Should they receive medicine?

What should they do with the medicine they receive?

The treatment for malaria infection is 3-days worth of pills. Should they finish the medicine?

Should they save the medicine?

Should they trade or sell the medicine for something they need?

How many people have you tested for malaria in the past year?

How many of those were during the test and treat campaign?

How many of those were just routine health worker duties?

When you give medicine to those testing positive, do those receiving the medicine finish it?

Why might someone not finish the medicine?

Do you consider that a problem?

Is it the same during the campaign as it is during regular duties?

What do you think about people receiving medicine for malaria infection when they test positive even if they are not sick with symptoms?

Do you know anybody who refused being tested for malaria infection, or who refused his or her child being tested for malaria infection?

Why might someone refuse a malaria infection test?

What do you do/say when someone refuses to be tested?

Do you know anybody who refused treatment for malaria infection, or who refused his or her child treatment for malaria infection?

Why might someone refuse treatment for a malaria infection?

What do you think about the campaign to screen everybody in your community for malaria infection?

What do other people think about the campaign?

Are people in your community refusing being tested for malaria infection?

Why might someone refuse being tested for malaria infection?

Are people in your community finishing their medicine when they test positive for malaria infection?

Why might someone not finish their medicine when they test positive for malaria infection?

How many times/often have you participated in the test and treat campaigns in your community?

How long does it take to complete a campaign in your community? Is it too long?

Have the campaigns affected your regular duties as a community health worker? How so?

Have the campaigns affected your relationship with the communities that you serve?

How so?

Would any changes make the campaigns better?

How long would you be able to continue working in these campaigns?

Have you learned anything about your communities from the campaign activities?

What have you learned?

Have you changed anything regarding your work as a CHW since the test and treat campaigns started?

What have you changed?

**Interview guide for group interviews with rural health centers, district health management teams, and ministry of health officials**

**(Research questions 3, 4, 5)**

What do you think about the test and treat campaigns in Southern Province?

Is the test and treat campaign something that you might consider applying in other areas?

What makes it appealing?

What makes it unappealing?

Would you conduct the campaigns any differently?

What changes might you make and why?

Have the campaigns had any other influence other than the actual intervention being conducted?

What do you think about the data coming back from the campaigns?

Has this data influenced your decision-making?

How can the data be accessed & used/what do you use the data for?

How are the campaigns affecting the community health workers' regular duties?

What do you think about the data coming back from the cell phones in rural health centers?

Has this data influenced your decision-making?
