# Supplementary material for: Assessment of the Acceptability of Testing and Treatment during a Mass Drug Administration Trial for Malaria in Zambia Using Mixed Methods
Source: Am J Trop Med Hyg. 2020 Jun 2;103(2 Suppl):28–36. doi: 10.4269/ajtmh.19-0663 (PMC7416978; doi:10.4269/ajtmh.19-0663)
Supplement: Supplementary file 4 [file tpmd190663.SD4.docx]

| **Description** | **Code** |
| --- | --- |
| 1. **Feelings**   **Happy (HAP)**  1.1Can receive treatment if found with malaria (Treatment)   - 1. Reduced distances to receive/access malaria health services (Reduced Distance)   2. Number of people getting sick from malaria reduced (Reduced Disease Burden)   3. People are protected from malaria when they take drugs even if they test negative for malaria (Protection from malaria)  1. **Reasons for taking treatment**   **2.1 Acceptance (ACC)**  **2.1.1**  People getting better after taking drugs (Healed by drugs)  2.1.2 Health services brought closer to community members  2.1.3 Reduced number of pills to be taken compared to coartem (Reduced pill burden)  2.1.4 Trust health workers (CHW), health system (Trust)  **2.2Refusals (REF)**  2.2.1 Refusals due to suspicions around Satanism (Satanism)  2.2.2 Refusals due to Inadequate information  2.2.3 Fear that blood collected will also be used for HIV diagnosis  2.2.4 Jealous of Community Health workers who were getting paid for doing MDA (Jealousy)  2.2.5 DHAp drug not administered through the health facility  2.2.6 People think it is not a government initiative but an NGO initiative  2.2.7 CHWs not taking drugs themselves  2.2.8 Inconsistent messaging, instructions | HAP-TRT  HAP- DIST  HAP- REDBUR  HAP- PRTED  ACC-HLD  ACC-DIST  ACC- RED_PILLS  ACC-TRST  REF- SAT  REF- INAD_INFO  REF- HIV_DIAG  REF-JLS_CHW  REF-DHAp_NOT_HF_ADMIN  REF- NOT GRZ_IN  REF- |
| **3. Treatment Adherence (TADH)**  3.1 Non Adherence due to inadequate information 3.2 Non Adherence due to fear of side effects  3.3 Non Adherence because one feels better  3.4 Non Adherence due to negative malaria test  3.5 Non Adherence due to DHAp pills perceived similar to ARV drugs  3,6 Adherence acceptance | ADH-INAD_INFO  ADH-SDE_EFTS  ADH- FEL_BETR  ADH-NGT_MAL_TST  ADH-ARVs_SIM_DHAp  ADH-ACC |
| **4. Recommendations for Malaria MDA campaigns (REC)**  4.1.1 Continuous supply of malaria drugs (DHAp)  4.1.2 Allocate more transport  4.1.3 change season for implementation from rainy season (Season)  4.1.4 Increased sensitization on the benefits of the MDA program  4.1.5 Re- orient /train community health workers | REC- CON_SUP_DHAp  REC- TRANS  REC-SESN  REC-SENSTZN  REC-CHW_RETRN |

| **5.CHW CHALLENGES**   - 1. Inadequate transport to go round communities   2. inadequate allowances   3. Rains making it difficult to reach places and find people in homes | CHA-INAD_TRPT  CHA-INAD_ALWCS  CHA-RAIN_SESN |
| --- | --- |
| **6.WORKLOAD HEALTH WORKERS**  6.1 Reduced number of people seeking treatment for malaria at the health facility due to treatment being provided in communities. | WORK-REDUCED |
